# Pharmacophore Based Virtual Screening Approach to Identify Selective PDE4B Inhibitors

**Published:** 2017

**Authors:** Anand Gaurav, Vertika Gautam

**Affiliations:** a *Faculty of Pharmaceutical Sciences, UCSI University, No. 1, UCSI Heights, Jalan Menara Gading, Taman Connaught, 56000 Kuala Lumpur, Federal Territory of Kuala Lumpur, Kuala Lumpur, Malaysia. *; b *Department of Chemistry, Faculty of Science, University of Malaya 50603, Kuala Lumpur, Malaysia.*

**Keywords:** Phosphodiesterase 4, Pharmacophore, HypoGen, Virtual screening, Zinc database, docking

## Abstract

Phosphodiesterase 4 (PDE4) has been established as a promising target in asthma and chronic obstructive pulmonary disease. PDE4B subtype selective inhibitors are known to reduce the dose limiting adverse effect associated with non-selective PDE4B inhibitors. This makes the development of PDE4B subtype selective inhibitors a desirable research goal. To achieve this goal, ligand based pharmacophore modeling approach is employed. Separate pharmacophore hypotheses for PDE4B and PDE4D inhibitors were generated using HypoGen algorithm and 106 PDE4 inhibitors from literature having thiopyrano [3,2-d] Pyrimidines, 2-arylpyrimidines, and triazines skeleton. Suitable training and test sets were created using the molecules as per the guidelines available for HypoGen program. Training set was used for hypothesis development while test set was used for validation purpose. Fisher validation was also used to test the significance of the developed hypothesis. The validated pharmacophore hypotheses for PDE4B and PDE4D inhibitors were used in sequential virtual screening of zinc database of drug like molecules to identify selective PDE4B inhibitors. The hits were screened for their estimated activity and fit value. The top hit was subjected to docking into the active sites of PDE4B and PDE4D to confirm its selectivity for PDE4B. The hits are proposed to be evaluated further using *in-vitro* assays.

## Introduction

Prevalence of inflammatory diseases of respiratory tract *i.e.* asthma and COPD has increased in recent years, with more than 200 million people affected by it worldwide. Most of the mortality related to these inflammatory disorders occurs in low and low-middle income countries (1). 

Phosphodiesterase 4 (PDE4), a predominant family of phosphodiesterase (PDE) enzymes expressed in immune and inflammatory cells, includes three subtypes PDE4A, PDE4B and PDE4D. Inhibition of PDE4 has been shown to suppress a diverse spectrum of inflammatory responses *in*-*vitro *and *in-vivo *(2-5). More importantly, many PDE4 inhibitors in development are efficacious in animal models of various inflammatory disorders, such as asthma, COPD, psoriasis, inflammatory bowel diseases, and rheumatoid arthritis (3, 6, 7), as well as in clinical trials for asthma and COPD (8-10).

The development of PDE4 inhibitors has been slowed down due to narrow therapeutic window of most of the compounds. A major reason for their poor clinical results is the consequence of dosing limitation caused by side effects such as nausea and emesis (11). Recent findings in PDE4D knockout mice suggest that an inhibitor with PDE4B selectivity should retain many beneficial anti-inflammatory effects without the unwanted side effects (12). The study also established that PDE4D inhibition is responsible for the dose limiting side effects. Some other studies have proven that selective PDE4B inhibitors have potent anti-inflammatory effects *in-vitro* and *in-vivo*. Investigation in ferrets also showed significantly less emesis with this compound compared with the non-selective PDE4 inhibitor cilomilast (13). Thus, PDE4B has been established as an extremely attractive target for design of anti-inflammatory drugs, particularly for asthma and COPD.

The highly conserved catalytic domain of PDE4 isozymes makes the design of inhibitors with PDE4 subtype selectivity a challenging task, nevertheless subtype selective PDE4 inhibitors have recently been described (14, 15). Only a few amino acids are non-conserved in N-terminal regulatory domain UCR2 (*i.e* Phe in PDE4D vs Tyr in PDE4B) and C-terminal domain CR3 (*i.e* Leu in PDE4D vs Gln in PDE4B) (16, 17). These minor differences in the regulatory domains have been exploited to design selective PDE4B or PDE4D inhibitors so far (16-18).

The availability of PDE4B and PDE4D inhibition data for recently reported PDE4 inhibitors allows the development of pharmacophore models of PDE4B and PDE4D inhibitors (19-21). Pharmacophore models also help in the identification of structural features which can differentiate between the two receptor subtypes. The information obtained can be used for design of more selective and potent PDE4B inhibitors with hitherto new structures. The pharmacophore models of PDE4B and PDE4D inhibitors can be used to screen databases of drug like compounds in a sequential manner to identify novel leads as selective PDE4B inhibitors. Pharmacophore model based virtual screening has proved to be a useful strategy for identification of novel leads in the past (22-32). In the present study pharmacophore models of both PDE4B and PDE4D inhibitors has been developed and validated. The pharmacophore models were then used for sequential virtual screening to identify novel selective PDE4B inhibitors. The hits were screened for their estimated activity and fit value. Their selectivity for PDE4B was confirmed by docking studies.

## Experimental

*Data set*

**Figure 1:**
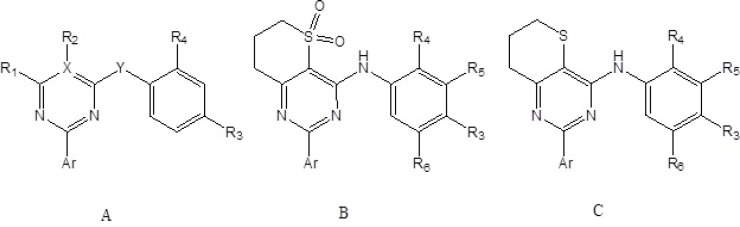
General structures of 2-arylpyrimidines (A), triazines (A) and thiopyrano[3,2-d]Pyrimidine (B and C).

Selective PDE4B inhibitors belonging to thiopyrano[3,2-d] Pyrimidines,2 arylpyrimidines and triazines class reported recently, along-with their PDE4B and PDE4D inhibitory activities, were used for the present study (Figure 1) (19-21). The molecular structures and IC_50_ of the above series were taken from the original papers. Numbers used in original papers were used to denote molecules belonging to triazine series while numbers used in original papers for molecules belonging to 2-arylpyrimidine and thiopyrano[3,2-d]Pyrimidine series were suffixed with a and b respectively.

*3D QSAR pharmacophore modeling*

*Pharmacophore generation*

Pharmacophore modeling is the most widely used method for identification of essential structural features required for biological activity. In the present study, HypoGen algorithm was applied to build the 3D QSAR pharmacophore models for both PDE4B and PDE4D inhibitors using DS V2.0 software (Accelrys Inc., San Diego, CA, USA) (33). 

For the study, 75 molecules, with activity values (IC_50_) between 3.0 nM and 18755 nM were selected as training set, which was used to engender the hypotheses. The training set selected complies with the requirements specified in the literature. To validate the hypothesis, the test set was prepared using the specified requirements. Test set contains 24 molecules having wide range of activity values. Sketch function of DS was used to sketch the two-dimensional (2D) chemical structures of all molecules which were later converted into 3D structures. Maximum of 250 conformations were generated for each molecule using the best conformation model generation method based on CHARMm force field and Poling algorithm (34). Those conformations with energy higher than 20 kcal/mol from the global minimum were rejected. Molecules with their conformational models were then submitted to DS for generating hypotheses.

**Figure 2:**
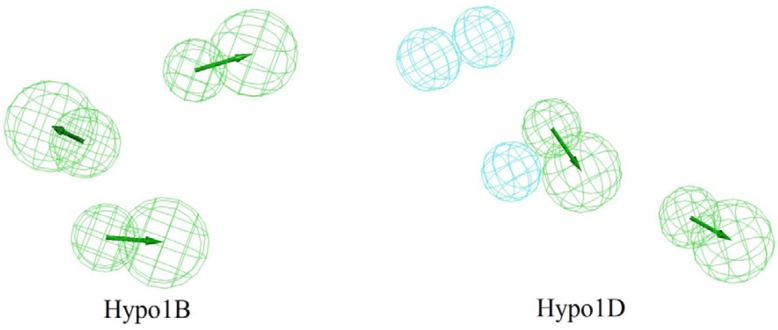
Hypo1B and Hypo1D chemical features with their geometric parameters

**Figure 3:**
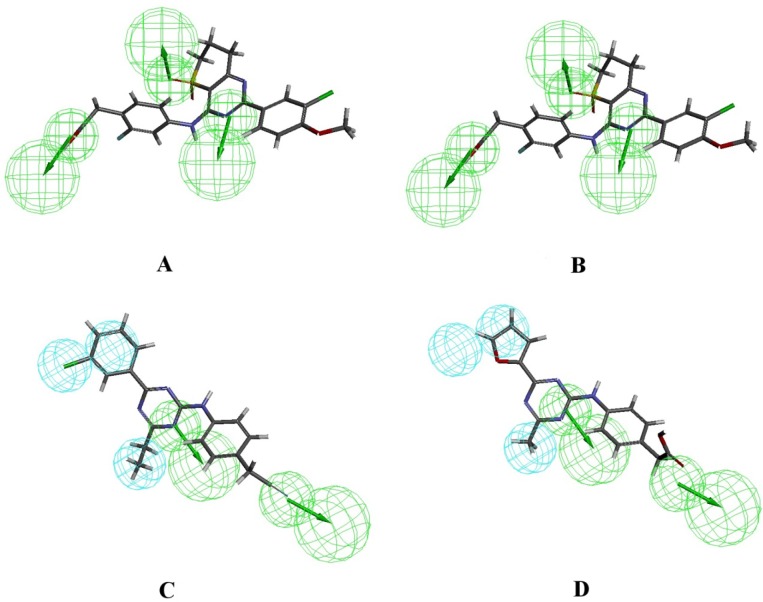
A. Most active PDE4B inhibitor (54b) aligned with Hypo1B, B. Least active PDE4B inhibitor (10) aligned with Hypo1B, C. Most active PDE4D inhibitor (29) aligned with Hypo1D, D. Least active PDE4D inhibitor (10) aligned with Hypo1D

**Figure 4:**
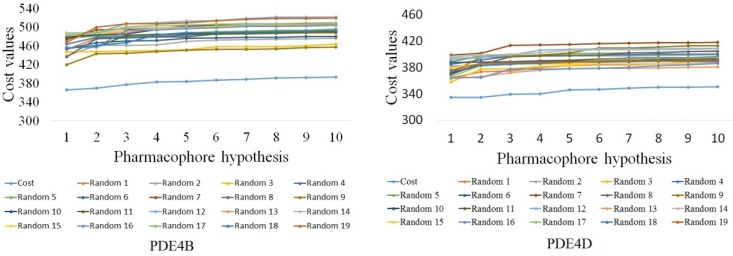
The difference in costs between HypoGen runs and the scrambled runs for PDE4B and PDE4D. The 95% confidence level was selected

**Figure 5:**
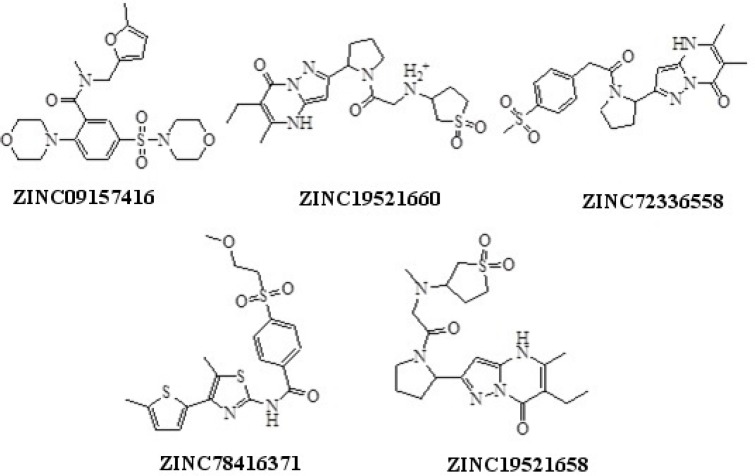
Structures of hits obtained using pharmacophore based virtual screening

**Figure 6.**
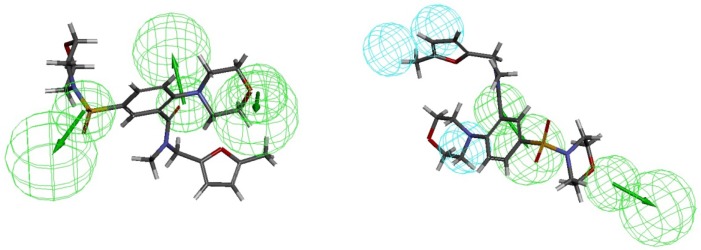
Most selective PDE4B inhibitor (ZINC09157416) identified by virtual screening aligned with Hypo1B and Hypo1D

**Table 1:** Information of statistical significance and predictive power presented in cost values measured in bits for the top 10 hypotheses as a result of automated 3D QSAR pharmacophore generation for PDE4B

**Hypo no.**	**Total cost**	**Cost difference** a	**RMS** b	**Correlation**	**Features** b	**Max fit**
Hypo1B	365.722	143.378	1.86	0.9571	HBA, HBA, HBA	5.8678
Hypo2B	369.858	139.242	1.89	0.9362	HBA, HBA, H	5.7607
Hypo3B	376.722	132.378	1.93	0.9208	HBA, HBA, H	5.2594
Hypo4B	382.828	126.172	1.97	0.9068	HBA, HBA, H, H	6.6554
Hypo5B	383.112	125.988	1.98	0.9054	HBA, HBA, H	5.2458
Hypo6B	386.676	122.424	1.99	0.8972	HBA, HBA, H	5.0619
Hypo7B	387.872	121.228	2.01	0.8933	HBA, HBA, H	5.4563
Hypo8B	391.358	117.742	2.03	0.8862	HBA, HBA, H	4.8644
Hypo9B	391.861	117.239	2.03	0.8858	HBA, HBA, H	4.6476
Hypo10B	393.315	115.785	2.04	0.8815	HBA, HBA, H	4.7971

a Cost difference between the null and the total cost. The values for null cost, fixed cost, and configuration cost are 509.10, 236.38, and 12.53 respectively.

b Abbreviations: RMS: root mean square deviation, HBA: hydrogen bond acceptor, HBD: hydrogen bond donor, H: hydrophobic.

**Table 2:** Information of statistical significance and predictive power presented in cost values measured in bits for the top 10 hypotheses as a result of automated 3D QSAR pharmacophore generation for PDE4D

**Hypo no.**	**Total cost**	**Cost difference** c	**RMS**	**Correlation**	**Features**	**Max fit**
Hypo1D	334.571	164.419	1.66	0.9563	HBA, HBA, H, H, H	8.1515
Hypo2D	335.16	163.830	1.66	0.9336	HBA, HBA, H, H	8.0525
Hypo3D	339.583	159.407	1.70	0.9247	HBA, HBA, H	6.0897
Hypo4D	340.037	158.953	1.67	0.8712	HBA, HBA, H, H,	5.6251
Hypo5D	346.347	152.643	1.74	0.8341	HBA, HBA, H, H, H	6.9568
Hypo6D	346.670	152.320	1.70	0.8027	HBA, HBA, H, H, H	4.5456
Hypo7D	348.692	150.298	1.76	0.7991	HBA, HBA, H, H	6.2123
Hypo8D	350.222	148.768	1.77	0.7892	HBA, HBA, H, H	6.7776
Hypo9D	350.437	148.553	1.76	0.7554	HBA, HBA, H, H, H	6.3886
Hypo10D	350.729	148.261	1.74	0.7332	HBA, HBA, H, H, H	4.8890

c The values for null cost, fixed cost and configuration cost are 498.99, 231.346 and 12.49 respectively.

**Table 3:** Actual and estimated activity of the training set molecules based on the pharmacophore model Hypo1B.

	**Fit value** ^d^	**LogIC**_50_	**LogIC**_50 _ **(predicted)**	**Error** ^e^
1	3.9094	8.8099	7.8080	1.0018
2	5.0896	4.0073	4.0906	-0.0832
4	4.4407	7.5730	6.5847	0.9884
5	4.5557	5.9480	5.3199	0.6282
8	4.2395	5.4681	6.0481	-0.5800
9	4.0517	8.2348	8.4804	-0.2455
10	3.9096	9.8392	8.8077	1.0316
12	3.9090	7.2862	6.8089	0.4773
14	4.0043	7.7328	6.5895	1.1433
15	4.4623	6.7393	5.5349	1.2045
18	4.6695	5.5645	5.0579	0.5066
19	3.8297	7.2399	6.9917	0.2483
20	4.5970	5.3936	5.2249	0.1688
22	4.1433	3.8286	4.2695	-0.4409
23	4.2621	4.9053	5.9959	-1.0906
24	3.9079	4.9053	5.8114	-0.9062
27	5.0484	5.4848	5.8855	-0.4007
28	4.5921	2.4849	2.2360	0.2489
29	4.8138	5.1060	4.7256	0.3804
31	4.6269	4.2341	4.1559	0.0782
32	5.1069	5.9965	5.6508	0.3457
33	4.6877	6.9939	6.0161	0.9779
10a	4.3958	5.7038	5.6880	0.0158
12a	3.9014	3.5264	4.8266	-1.3002
13b	4.6589	4.0431	4.0824	-0.0393
14a	4.0112	6.5367	6.5736	-0.0369
14b	5.2346	4.7875	4.9567	-0.1692
15a	4.6777	4.7875	5.0390	-0.2515
16b	4.6630	3.4012	4.0729	-0.6717
17a	4.6442	7.9374	7.1161	0.8213
17b	5.4607	2.3979	2.5361	-0.1382
18a	3.8934	8.2161	7.8450	0.3711
18b	4.9305	4.7875	4.4568	0.3307
19a	4.0354	6.7569	6.5180	0.2390
1b	5.6617	3.2189	3.4733	-0.2544
20b	5.1383	3.5264	3.9785	-0.4522
21a	3.9115	5.3471	6.8032	-1.4561
21b	4.9923	4.9416	4.3147	0.6270
22a	5.0677	5.3936	5.5410	-0.1474
22b	5.1410	4.9416	4.9722	-0.0305
23a	4.6139	5.0106	5.1859	-0.1753
24b	4.5382	6.0638	5.3603	0.7035
26a	3.9114	6.8459	6.8035	0.0424
26b	5.4570	3.7842	3.5446	0.2396
27a	3.9116	7.0901	6.8031	0.2870
27b	5.5718	3.6376	3.9802	-0.3426
28b	5.3524	2.3026	2.4854	-0.1829
29a	5.1079	5.7683	5.0485	0.7198
29b	5.4456	5.5215	5.2709	0.2506
2a	3.8825	6.0638	6.8700	-0.8062
2b	5.0910	3.3673	3.2873	0.0800
30a	5.1379	5.2470	4.6793	0.5678
31a	5.4163	3.5264	3.3383	0.1881
31b	5.5135	3.8286	3.5145	0.3142
32b	5.5108	2.5650	2.3208	0.2442
33a	5.0896	2.7081	2.4906	0.2175
33b	5.5519	2.9444	3.0262	-0.0817
34a	5.1824	1.9169	1.8768	0.0401
35a	5.2004	2.7081	2.4355	0.2725
35b	5.4536	3.4012	3.2525	0.1487
36b	5.5615	2.6391	3.0039	-0.3649
37b	5.5723	2.1163	2.3791	-0.2628
39b	5.6194	2.3026	2.8707	-0.5681
3a	3.9080	4.9416	4.8113	0.1303
3b	4.8032	3.0910	4.7500	-1.6590
44b	5.5305	1.5261	2.0753	-0.5493
45b	5.6535	2.7726	2.7922	-0.0196
47b	5.6493	4.1589	3.8018	0.3571
48b	5.4860	3.8918	3.5778	0.3140
49b	5.6225	2.2925	2.8636	-0.5711
4a	3.9099	7.1701	6.8070	0.3632
53b	5.5256	2.1041	2.0866	0.0176
54b	5.7574	1.0986	1.3529	-0.2542
55b	5.7462	1.7750	1.5787	0.1962
8a	4.0575	4.7875	5.4670	-0.6795

**Table 4:** Actual and estimated activity of the training set molecules based on the pharmacophore model Hypo1D

	**Fit value**	**LogIC**_50_	**LogIC**_50 _ **(predicted)**	**Error**
1	5.8033	7.7407	7.1109	0.6297
2	5.7524	7.5994	7.2281	0.3713
4	6.3846	8.1831	8.7724	-0.5893
5	5.8364	6.7627	7.0346	-0.2718
8	5.9387	7.0741	6.7991	0.2751
9	5.4415	8.6325	8.9440	-0.3115
10	5.5496	10.0105	9.6951	0.3154
12	5.8037	6.5889	7.1100	-0.5211
14	5.7043	6.7558	7.3388	-0.5830
15	6.1765	6.5876	6.2514	0.3361
18	5.9054	5.5255	5.8757	-0.3502
19	5.6042	5.3327	5.5694	-0.2367
20	5.9951	5.3891	5.6693	-0.2803
22	6.9164	2.9957	2.5479	0.4478
23	5.6015	5.5910	5.5755	0.0155
24	5.9404	5.5910	5.7952	-0.2042
27	6.7011	5.3613	5.0437	0.3176
28	7.6438	2.5650	2.8729	-0.3079
29	7.6555	1.9459	1.8460	0.0999
31	6.9078	4.1431	4.5677	-0.4246
32	6.6422	5.0752	5.1791	-0.1039
33	6.0901	4.5644	4.4505	0.1138
10a	5.7511	7.3132	7.2311	0.0822
12a	5.6731	4.4067	4.4106	-0.0039
13b	5.6202	6.5221	6.5325	-0.0104
14a	5.5703	7.7832	7.6474	0.1358
14b	5.7270	6.9078	7.2865	-0.3788
15a	5.6576	7.1701	7.4463	-0.2761
16b	5.7635	5.6699	5.2026	0.4673
17a	5.9350	9.3927	9.8075	-0.4149
17b	5.8604	5.6348	5.9794	-0.3446
18a	5.6062	9.6158	9.5646	0.0512
18b	5.9060	7.4955	7.8744	-0.3788
19a	4.8642	8.0064	8.2731	-0.2668
1b	5.9002	7.9374	7.8878	0.0496
20b	5.6507	6.9078	6.4623	0.4455
21a	6.0462	7.3132	7.5516	-0.2384
21b	5.6546	8.9092	8.4532	0.4560
22a	5.8046	7.6009	7.1079	0.4930
22b	5.8002	7.3778	7.1180	0.2598
23a	5.8689	7.1701	6.9599	0.2102
24b	5.7926	6.0638	6.1355	-0.0717
26a	5.7676	9.3057	9.1931	0.1125
26b	5.8920	7.3132	6.9066	0.4066
27a	5.8319	9.2003	9.0450	0.1553
27b	5.9663	7.6497	7.7356	-0.0859
28b	5.8525	6.7569	6.9975	-0.2406
29a	5.8267	9.0825	9.0570	0.0255
29b	5.9607	8.2428	8.7484	-0.5056
2a	4.6529	8.1315	8.7597	-0.6281
2b	5.5577	7.1701	7.6763	-0.5062
30a	5.7913	8.5755	8.1385	0.4370
31a	5.7056	7.3132	7.3358	-0.0225
31b	5.9145	8.1315	8.8549	-0.7233
32b	5.9184	7.1701	7.8458	-0.6757
33a	5.7524	7.4384	7.2281	0.2103
33b	5.9296	6.2538	6.8200	-0.5662
34a	5.8837	7.9725	7.9258	0.0467
35a	5.7404	8.0392	7.2558	0.7834
35b	5.8956	5.6699	6.8983	-1.2284
36b	5.9438	6.0868	6.7874	-0.7006
37b	5.8532	5.9915	6.9959	-1.0044
39b	5.9199	6.9078	6.8424	0.0654
3a	5.6610	7.6497	7.4385	0.2112
3b	5.9294	5.5607	6.8206	-1.2599
44b	5.9348	6.4297	6.8080	-0.3782
45b	5.9371	7.1701	6.8027	0.3675
47b	5.9539	7.1701	6.7642	0.4059
48b	5.9414	6.3969	6.7930	-0.3960
49b	5.8956	6.3279	6.8984	-0.5704
4a	5.5735	8.9359	7.6399	1.2960
53b	5.9012	7.2442	6.8854	0.3588
54b	5.8107	7.1701	7.0938	0.0763
55b	5.9243	6.6970	6.8322	-0.1352
8a	5.7105	7.3132	7.3247	-0.0114

**Table 5:** Actual and estimated activity of the test set molecules based on the pharmacophore model Hypo1B

	**Log (Activ)**	**Log (Estimate)**	**Error**
7	5.5255	5.3455	0.1800
11	7.2703	7.2345	0.0358
16	7.5294	7.4567	0.0727
21	9.0842	7.3563	1.7279
26	6.6606	5.3455	1.3151
30	4.8363	3.8355	1.0008
34	7.3614	7.3253	0.0361
11a	6.2916	5.7354	0.5562
16a	4.2195	4.3323	-0.1128
20a	5.3936	5.3452	0.0484
24a	4.3567	4.9752	-0.6185
28a	6.3969	5.3453	1.0516
32a	2.9444	2.9968	-0.0524
1a	5.2470	4.8659	0.3811
15b	4.7875	5.8364	-1.0489
19b	3.6376	4.7264	-1.0888
23b	4.9416	3.8563	1.0853
30b	4.0254	4.3324	-0.3070
34b	3.6376	3.7254	-0.0878
38b	2.9957	3.2232	-0.2275
46b	1.6487	2.8675	-1.2188
52b	2.0149	2.2484	-0.2335
56b	1.3350	2.4543	-1.1193
12b	6.7799	7.2194	-0.4395

**Table 6:** Actual and estimated activity of the test set molecules based on the pharmacophore model Hypo1D

	**Log (Activ)**	**Log (Estimate)**	**Error**
7	7.3059	7.1533	0.1526
11	7.6530	7.9863	-0.3333
16	7.7267	7.2121	0.5146
21	6.7867	6.9891	-0.2024
26	6.5236	6.3334	0.1902
30	4.8828	4.6276	0.2552
34	6.3969	6.6676	-0.2707
11a	8.9872	8.2223	0.7649
16a	6.8977	6.5122	0.3855
20a	7.9374	7.2231	0.7143
24a	6.6333	6.9098	-0.2765
28a	8.4764	8.2957	0.1807
32a	7.3778	7.5762	-0.1984
1a	7.5496	7.8894	-0.3398
15b	6.0403	5.7204	0.3199
19b	6.6720	6.8732	-0.2012
23b	7.6962	7.9909	-0.2947
30b	7.0901	6.7925	0.2976
34b	6.6720	6.7623	-0.0903
38b	7.0031	7.4052	-0.4021
46b	6.3630	6.8437	-0.4807
52b	7.1701	7.4923	-0.3222
56b	6.3969	6.5427	-0.1458
12b	8.1315	8.4072	-0.2757

**Table 7:** Fit values of hits with Hypo1B and Hypo1D

	**Fit value (Hypo1B)**	**Fit value (Hypo1D)**
ZINC09157416	4.38886	0.13199
ZINC19521660	4.33458	0.74828
ZINC72336558	4.33584	0.22303
ZINC78416371	4.50232	0.52406
ZINC19521658	4.36043	0.40399

**Table 8.** CDOCKER energy of hits and standard (33b) with PDE4B and PDE4D

	**-CDOCKER energy (PDE4B)**	**-CDOCKER energy (PDE4D)**
34b	-13.0258	-22.3445
ZINC09157416	-15.9889	-26.7976

Automated 3D QSAR pharmacophores were produced by comparing the PDE4B and PDE4D inhibitory activity values of molecules in the training set separately. This helps in identifying the features that are common with the active compounds, but excludes common features for the inactive compounds within conformational allowable regions of space. Selecting the chemical features is one of the most important steps in generating a pharmacophore. While generating hypotheses, HBA (hydrogen bond acceptor), HBD (hydrogen bond donor), and H (hydrophobic), features were selected based on the training set molecules. The number of features allowed in the model were kept in the range 0-5. The ‘Uncertainty’ values for all the 75 molecules in the training set were set to 2.0, and the default values for other parameters were kept constant. Subsequently, pharmacophore models were computed and the 10 top scoring hypotheses for both PDE4B and PDE4D inhibition were selected for further study. The qualities of the hypotheses were reliant on the fixed cost, null cost, and total cost values (35).

*Assessment of pharmacophore quality*

Quality of the developed pharmacophore was assessed using three different methods. Initially, cross validation was performed by the Fischer’s randomization test. Secondly, the prediction of the activity values of the test set was performed. The correlation between the experimental and predicted activities was used to assess predictive ability of the model. All queries were addressed using the ligand pharmacophore mapping protocol. 

*Virtual Screening*

The validated pharmacophore model (Hypo1B and Hypo1D) of PDE4B and PDE4D inhibitors was used as a query in a sequential manner to search the zinc database. Zinc is a comprehensive database of small molecules containing a total of 17,900,742 drug like molecules (36). In the first step ligand pharmacophore mapping module of DS was used along with Hypo1B as the pharmacophore model and zinc database as the database. In the next step, hits mapping to the pharmacophore model Hypo1B were retrieved and hit compounds showing Hypo1B estimated IC_50 _less than 20 nM were selected and subsequently subjected to screening using the validated pharmacophore model Hypo1D in the same manner as in the previous step. The hit compounds were chosen that showed Hypo1D estimated fit value less than 4. 

Docking studies were used to confirm the selectivity of the hits obtained using the pharmacophore based virtual screening. The most PDE4B selective hit determined by the fit values for Hypo1B and Hypo1D and the most selective ligand from the series used for pharmacophore development *i.e.* 34b, were docked into the active sites of PDE4B (PDB ID: 4NW7) and PDE4D (PDB ID: 1Y2B). First the protein structures were prepared using the automatic protein preparation module of DS V2.0 software using the default parameters. The structures of the identified hit as well as the standard molecule (34b) were prepared using the prepare ligand module of DS V2.0 software. Docking of the prepared ligands into the active site of the prepared structures of PDE4B and PDE4B was carried out using CDOCKER program available in DS V2.0 software (Accelrys Inc., San Diego, CA, USA) with default parameters (37). The ratio of PDE4B/PDE4D docking scores was used as measure of PDE4B selectivity. The higher is the ratio the greater is the PDE4B selectivity. 

## Results and Discussion

*3D QSAR pharmacophore modeling*

*Pharmacophore generation*

The top scoring model (Hypo1B) for PDE4B inhibition consist of three HBA which established the highest cost difference (143.378), best correlation coefficient (0.9571), maximum fit value (5.8678) and lowest root mean square (RMS) of 1.86 (Table 1). The results revealed the importance of HBA in PDE4B receptor antagonist activity. The fixed and the null cost values were 236.38 and 509.10, respectively (Table 1). Difference between these two costs (143.378) was greater than 70 bits which showed that the model has over 90% statistical significance. A good pharmacophore model should also have the configuration cost lower than 17, and it was found to be 12.53 for the generated pharmacophore hypotheses. Hypo1B showed correlation coefficient value of 0.9571, demonstrating its good prediction ability.

Top scoring model (Hypo1D) for PDE4D inhibition consists of two HBA and three H with highest cost difference (164.419), best correlation coefficient (0.9563), maximum fit value (8.1515), and lowest root mean square (RMS) of 1.66 (Table 2). As in the case of Hypo1B, HBA was found to be important for PDE4D receptor antagonist activity although there is additional H in this case. Difference between fixed and null costs (164.419) showed that the model has over 90% statistical significance. The configuration cost was also sufficiently low at 12.49. Hypo1D showed correlation coefficient value of 0.9563 (Table 2). Based on statistical parameters Hypo1B and Hypo1D were selected as the best hypothesis for PDE4B and PDE4D inhibition respectively and were employed for further analyses. 

Figure 2 shows Hypo1B, and Hypo1D chemical features with their geometric parameters while Molecules with highest and lowest activity in the training set aligned to Hypo1B and Hypo1D are shown in Figure 3. The prediction accuracy of both the models was verified using the training set and the activity of each molecule in training set was estimated by regression analysis.

The experimental and predicted activities by Hypo1B and Hypo1D for 75 training set molecules are shown in Tables 3 and 4 respectively. Data clearly shows the good agreement between predicted and experimental IC_50_ values.

^d^Fit value indicates how well the features in the pharmacophore overlap the chemical features in the molecule. Fit value = weight x [max(0,1 - SSE)] where SSE = (D/T)^2^, D = displacement of the feature from the center of the location constraints and T = the radius of the location constraint sphere for the feature (tolerance).

^e^Difference between the predicted and experimental values. ‘+’ indicates that the predicted IC_50_ is higher than the experimental IC_50_; ‘-’ indicates that the predicted IC_50_ is lower than the experimental IC_50_; a value of 0 indicates that the predicted IC_50_ is equal to the experimental IC_50_.

Close examination of the pharmacophore models Hypo1B and Hypo1D reveals the structural features of an inhibitor which can differentiate well between the two receptors. The conformation which can allow –COOH at R_3_ and hydrophobic groups like halogen atoms in the aromatic ring (Ar) to orient properly for interaction with CR3 will show significant selectivity for PDE4B as compared to PDE4D. This is consistent with the findings described previously in the original papers in which these compounds have been reported (21). 

*Validation of Hypo1B and Hypo1D*

The generated hypotheses were validated using standard methods to check whether the best hypotheses are statistically significant and have considerable predictive ability.

*Fischer’s randomization method*

Fischer’s randomization was used to evaluate the statistical significance of the Hypotheses. Validation was done by generating random spreadsheets for training set molecules, which randomly reassigned activity values to every molecule and subsequently generated the hypotheses using the same features and parameters originated for Hypo1B and Hypo1D. All the randomly generated spreadsheets had higher total cost values and lower correlation coefficient values as can be seen clearly from Figure 4. This suggests that Hypo1B and Hypo1D were not generated by chance.

*Test set*

Test set was prepared using the same protocol as training set and used to determine whether the hypotheses were able to predict the active molecules other than those present in the training set.

The correlation coefficient (r) for the test set given by Hypo1B was 0.8579 (Table 5) while that by Hypo1D was 0.8299 (Table 6). Test set molecules were classified using the same criteria as used for training set molecules. Thus Hypo1B and Hypo1D were able to estimate the PDE4B and PDE4D inhibition activities respectively with reasonable accuracy.

*Virtual Screening*

Zinc, a comprehensive database of small drug like molecules was used for the sequential virtual screening using the pharmacophore models. Screening of zinc database using the validated pharmacophore model (Hypo1B) of PDE4B inhibitors retrieved a set of 6015 hits, mapping to the pharmacophore model Hypo1B. The hits comprised of some compounds structurally similar to that of the existing PDE4B inhibitors, and some novel scaffolds. 

The 397 hit compounds showing Hypo1B estimated IC_50 _less than 20 nM were selected and subsequently subjected to screening using the validated pharmacophore model Hypo1D. 5 hit compounds that showed Hypo1 PDE4D estimated fit value less than 4 were identified (Figure 5). Among the hits ZINC09157416 demonstrated the best PDE4B selectivity based on the hit values (Table 7). ZINC09157416 aligned with Hypo1B and Hypo1D is shown in Figure 6. 

The results of docking studies of ZINC09157416 and 34b with PDE4B and PDE4D further confirmed the selectivity of ZINC09157416 for PDE4B over PDE4D (Table 8).

## Conclusions

Ligand-based pharmacophore models for a diverse class of PDE4B and PDE4D inhibitors were developed. The best pharmacophore models Hypo1B and Hypo1D were validated using different methods to evaluate their predictive power over the diverse test set compounds. Hydrogen bond acceptors were identified to be mainly responsible for PDE4B inhibition while both hydrogen bond acceptors as well as hydrophobic groups were found to be responsible for PDE4D inhibition. The highly predictive pharmacophore hypotheses were further used in sequential virtual screening for identification of selective PDE4B inhibitors. Zinc drug like database was used in virtual screening. The hits from the virtual screening were filtered based on the estimated activity and fit value. Five molecules with different backbones were identified as final hits. The most selective hit molecule ZINC09157416 exhibited better selectivity for PDE4B than the standard compound 34b in the docking studies. The activity of the hit compound has not been reported in the literature as we explored by PubChem and SciFinder Scholar search tools. Thus, the sequential virtual screening strategy using 3D QSAR pharmacophores for PDE4B and PDE4D inhibitors proved to be an effective strategy to identify novel selective PDE4B inhibitors.
